# Selection of percutaneous aortic valve replacement candidates: CMR assessment of aortic valve stenosis and aortic root morphology in comparison with echocardiography and cardiac catheterization

**DOI:** 10.1186/1532-429X-11-S1-P96

**Published:** 2009-01-28

**Authors:** Bernard P Paelinck, Delphine Petillot, Christiaan J Vrints, Johan M Bosmans

**Affiliations:** University Hospital Antwerp, Edegem, Belgium

**Keywords:** Aortic Valve, Aortic Stenosis, Cardiac Catheterization, Aortic Root, Aortic Ring

## Introduction

Percutaneous aortic valve replacement in patients presenting high risk for surgery is a promising new interventional treatment modality. The potential role of non-invasive imaging techniques in patient selection needs further validation.

## Purpose

We aimed to compare 1. planimetry of aortic valve area (AVA) by CMR with 3D echocardiography and calculated AVA by Doppler and cardiac catheterization 2. aortic root dimensions by CMR with echocardiography and angiography.

## Methods

Twenty-eight high risk elderly symptomatic patients with severe aortic stenosis scheduled for potential percutaneous aortic valve replacement, were studied. AVA was determined using steady state free precession CMR and direct planimetry using 3D echocardiography. AVA was also calculated by cardiac catheterization using the Gorlin equation and by Doppler using the continuity equation. Diameter of aortic ring, sinus and sinotubular junction were measured using steady state free precession CMR, 2D echocardiography and invasive aortography.

## Results

Mean differences and 95% CI in AVA were 0.02 cm^2^ (-0.04, 0.08) (p = NS) for catheterization versus Doppler echocardiography, -0.01 cm^2^ (-0.08, 0.06) for catheterization versus 3D echocardiography (p = NS) and 0.01 cm^2^ (-0.07, 0.08) for catheterization versus CMR (p = NS).

Mean differences and 95% CI for diameter aortic ring, sinotubular junction and aortic sinus are displayed in table 1 (*p < 0.05).

**Table 1:** Aortic root dimensions

	Mean difference 2D echocardiography versus	
	invasive	CMR
Ring (cm)	0.42 (0.29, 0.55)*	-0.05 (-0.12, 0.02)
Sinus (cm)	0.03 (-0.10, 0.17)	-0.10 (-0.20, 0)*
Sinotubular junction (cm)	0.03 (-0.09, 0.15)	-0.13 (-0.23, -0.03)*

## Conclusion

1. CMR planimetry, Doppler and 3D echocardiography provided an accurate estimate of AVA in comparison with catheterization.

2. Catheterization underestimates aortic ring dimensions, while CMR overestimates aortic sinus and sinotubular junction dimensions in comparison with echocardiography.

